# Notes and News

**Published:** 1902-01-11

**Authors:** 


### Notes and News.

From the Birmingham Hospital Sunday Fund, the General Hospital in that city, has received a contribution this year of £5,000.

The Harben lectures of the Royal Institute of Public Health will be delivered in English by Dr. M. Gruber, of Vienna, the subject being the antibodies of the blood. The lectures will be delivered at King's College on January 13th, 14th, and 15th, at 5 p.m.

The study of physiology in the University of London should certainly be encouraged by Mr. Walter Palmer's liberality in providing scientific apparatus whereby the staff of voluntary lecturers will be enabled to offer suitably illustrated lectures to advanced students. This generosity on the part of Mr. Palmer and the lecturers is to be admired, but we question whether a free educational gift is ever appreciated at its proper value.

An excellent feature has been opened at the West Kirby Convalescent Home for Children. This home receives a number of chronic or protracted cases, and for some time past some ladies interested in the institution have been holding classes for the instruction of the little inmates. So useful have these proved that the Liverpool School Board have consented to provide education on a permanent basis, and have consequently provided a teacher and opened a school in connection with the home. The education will, of course, be adapted to the physical condition of the pupils.

Recent sanitary statistics from Madrid show that city to have a very unsatisfactory death-rate. This is largely attributed to over-crowding. The authorities are waking up, and one recent regulation might be adopted in our towns and health resorts with great advantage. This regulation provides for the fumigation of houses or apartments let on hire, by the municipal authorities. The risks run by those hiring dwellings or apartments would be minimised if the owners were obliged to produce a certificate of a reasonably recent date attesting to the satisfactory sanitary condition of the habitation.

The General Court of the German Hospital will be held on Friday, 31st inst., at two o'clock, at Winchester House, E.C.

King Edward's Hospital Fund for London has received for the Coronation gift a donation of £402 16s. from the directors, managers, and employés of the Gordon Hotels Company.

A meeting will be held at the Mansion House on Wednesday, January 15th, at three o'clock, in support of the appeal now being made on behalf of Guy's Hospital, when the Lord Mayor will preside.

A new hospital has been opened at Sutton, in Surrey, the gift of Mr. Passmore Edwards. The site has been given by Mr. R. C. Forster, of Sutton. The hospital has accommodation for 12 patients, and cost £3,000.

Colonel Sir T. J. Gallwey, Principal Medical Officer of the Home District, has been selected for appointment as Principal Medical Officer in India, with the rank of Surgeon-General, succeeding Surgeon-General W. Taylor, appointed Director-General of the Army Medical Service.

The highest number of small-pox cases reached in one day was recorded on the last day of the old year, and amounted to 42. Local authorities are adopting more stringent measures, and the Metropolitan Asylums Board is evidently determined to be found ready to meet any emergency. The recent fire on board the hospital ship really occurred in administrative quarters, and was promptly suppressed.

A medical paper in New York recently commented upon the surprising fact that a patient remained in the wards of a hospital with the first symptoms of small-pox upon him for three days. But if this is possible, how easy for small-pox patients to spread the disease amongst those with whom they associate before the presence of the disease is detected. During the present epidemic characteristic symptoms should be made universally known.

Experience in America shows the open-air treatment of phthisis to be as satisfactory as it is in Europe. It is fortunate that so much more confidence is placed in the treatment than the actual locality, as, otherwise, the keenest disappointment would have been felt by sufferers who are no longer allowed to land in America. At one time the Rocky Mountains and other American health resorts were regarded as effecting cures not possible elsewhere. Now most consumptives prefer to remain nearer home.

Mr. Henry Duncalfe, of Sutton Coldfield, near Birmingham, has bequeathed £5,000 to form a "Duncalfe Benevolent Fund," in connection with the Birmingham Medical Benevolent Society, and £5,000 to form a similar trust for the Royal Medical Benevolent College, the income of this latter trust to benefit the unmarried daughters of medical men. He has also left various sums of money to the General Hospital, Eye Hospital, the Queen's Hospital, the Orthopædic Hospital, and Blind Asylum, at Birmingham, and to the Llandudno Sanatorium, the Llanfairfechan Convalescent Home for Men, the Royal Alexandra Hospital at Rhyl, and the Home for Incurables, Leamington.

